# Postoperative Weight Loss After Antiobesity Medications and Revision Risk After Joint Replacement

**DOI:** 10.1001/jamanetworkopen.2024.61200

**Published:** 2025-02-21

**Authors:** Dongxing Xie, Martin Englund, Nancy E. Lane, Yuqing Zhang, Xiaoxiao Li, Jie Wei, Chao Zeng, Guanghua Lei

**Affiliations:** 1 Department of Orthopaedics, Xiangya Hospital, Central South University, Changsha, China; 2 Hunan Key Laboratory of Joint Degeneration and Injury, Changsha, China; 3 Key Laboratory of Aging-related Bone and Joint Diseases Prevention and Treatment, Ministry of Education, Xiangya Hospital, Central South University, Changsha, China; 4 Department of Clinical Sciences Lund, Orthopaedics, Clinical Epidemiology Unit, Lund University, Lund, Sweden; 5 Center for Musculoskeletal Health and Department of Medicine, University of California School of Medicine, Sacramento; 6 Division of Rheumatology, Allergy, and Immunology, Department of Medicine, Massachusetts General Hospital, Harvard Medical School, Boston; 7 The Mongan Institute, Massachusetts General Hospital, Harvard Medical School, Boston; 8 Department of Epidemiology and Health Statistics, Xiangya School of Public Health, Central South University, Changsha, China; 9 National Clinical Research Center for Geriatric Disorders, Xiangya Hospital, Central South University, Changsha, China

## Abstract

**Importance:**

The 2023 American College of Rheumatology and American Association of Hip and Knee Surgeons Clinical Practice Guideline concluded that obesity alone should not delay joint replacement. Therefore, a substantially increased utilization of joint replacement among patients with obesity could be expected. However, patients with obesity are at increased risk of revision, posing unique challenges as the surgery is complex and costly, and it remains unknown whether postoperative weight loss could decrease the risk of revision.

**Objective:**

To examine the association of the proportion of postoperative weight loss following antiobesity medication use with the risk of revision among patients with obesity undergoing hip or knee replacement.

**Design, Setting, and Participants:**

Using a target trial emulation, a causal inference framework, this retrospective cohort study investigated patients with obesity who underwent hip or knee replacement. Data were from the IQVIA Medical Research Database (2000-2023). Statistical analysis was performed from October 2023 to June 2024.

**Main Outcomes and Measures:**

Emulated analyses of a hypothetical target trial were assessed for the association of small-to-moderate (2%-10%) or large (≥10%) weight loss after initiating antiobesity medications (orlistat, sibutramine, glucagon-like peptide-1 receptor agonists, and rimonabant) within 1 year with the risk of 5-year and 10-year revision after initiation of antiobesity medications.

**Results:**

Among 3691 qualified participants (mean [SD] age, 64.7 [9.3] years; 2322 [62.9%] women), the 5-year risks of revision were 5.6%, 4.4%, and 3.7% for weight gain or stable, small-to-moderate weight loss, and large weight loss groups, respectively. Compared with the weight gain or stable group, the hazard ratios (HRs) were 0.75 (95% CI, 0.55-1.04) for the small-to-moderate weight loss group and 0.57 (95% CI, 0.36-0.91) for the large weight loss group. Similar results were observed when the analyses were performed separately for hip or knee replacement. The HRs for revision were 0.55 (95% CI, 0.32-0.93) for small-to-moderate weight loss and 0.49 (95% CI, 0.25-0.97) for large weight loss groups compared with the weight gain or stable group in patients undergoing knee replacement; the corresponding HRs for revision were 0.82 (95% CI, 0.54-1.25) and 0.53 (95% CI, 0.30-0.93) in patients undergoing hip replacement. Consistent findings were obtained regarding the association of weight loss with the 10-year risks after initiating antiobesity medications.

**Conclusions and Relevance:**

In this cohort study using a target trial emulation, a higher proportion of weight loss after initiating antiobesity medications within 1 year was associated with a lower risk of 5-year and 10-year revision among patients with obesity undergoing joint replacement. These results suggest that antiobesity medication use, with relatively safe and sustainable weight loss, may be an effective strategy for improving implant survivorship of hip and knee replacements in the obese population.

## Key Points

**Question:**

What is the association between postoperative weight loss with antiobesity medication use and the risk of revision in patients with obesity undergoing knee or hip replacements?

**Findings:**

In this cohort study, a higher proportion of weight loss after initiating antiobesity medications within 1 year was associated with a lower risk of 5-year and 10-year revision among patients with obesity undergoing hip or knee replacement.

**Meaning:**

These results suggest that pharmacotherapy, with relatively safe, effective, and sustainable weight loss, could be considered a strategy for improving implant survivorship of hip and knee replacements in the obese population.

## Teaser

This cohort study investigates whether postoperative weight loss with antiobesity medications is associated with risk of surgical revision among patients with obesity undergoing hip or knee replacement.

## Introduction

Hip and knee replacements are among the most common and successful elective surgical procedures.^1,2^ As the global population ages, rates of joint replacements are steadily rising, and this trend is expected to continue.^3^ In the United States, the current annual demand for hip replacement is approximately 400 000 cases, and knee replacement is around 900 000 cases.^4^ Although the outcome of these replacements is generally favorable, some prostheses may fail over time, leading to revision surgery, which is complex and can be costly.^5^ The rising demand for primary joint replacement inevitably leads to an increased need for revisions. The annual revision rate for hip and knee replacement is around 1%,^6,7^ and the cumulative risk of revision over 10 years is approximately 5%.^5,8^ Thus, identifying risk factors, particularly modifiable ones, for revision and exploring targeted interventions could enhance the longevity of joint replacements.

Obesity has become an epidemic, affecting 800 million people worldwide.^9^ Meanwhile, the population undergoing hip and knee replacements has become increasingly obese amidst this epidemic.^10^ However, studies have demonstrated that patients with obesity undergoing joint replacement surgical procedures are at 2 to 3 times higher risk of postoperative complications, including any infection or deep infection requiring surgical debridement, which could lead to revision surgical procedures.^11,12,13,14^ Yet, whether weight loss after surgery could mitigate this risk remains unclear. Considering that joint replacement is still a cost-effective and effective treatment for patients with morbid obesity experiencing severe pain and activity limitations due to end-stage osteoarthritis (OA),^15^ the 2023 Clinical Practice Guideline by the American College of Rheumatology and American Association of Hip and Knee Surgeons (ACR/AAHKS) established that obesity alone should not delay joint replacement.^16^ With the obesity rate among patients undergoing joint replacement likely to rise further, evaluating the outcomes of postsurgery weight loss becomes imperative, as it may offer an effective means to improve implant longevity among patients with obesity undergoing joint replacement.

To fill this knowledge gap, we conducted a population-based cohort study using a target trial emulation. We examined the association of the proportion of weight loss over 1 year after initiating antiobesity medications to the risk of revision after hip and knee replacements, as pharmacotherapy represents a relatively safe obesity treatment that can yield satisfactory weight loss outcomes and maintenance.^9^

## Methods

### Data Source

We used the IQVIA Medical Research Database (IMRD) data, incorporating The Health Improvement Network (THIN), a Cegedim database. IMRD contains longitudinal nonidentified patient electronic health care records collected from general practitioner (GP) clinical systems in the UK. IMRD includes data from more than 17 million patient records. The database holds patient demographic information, anthropometric characteristics, lifestyle factors, and details from visits to GPs. The Read classification system is used to code specific diagnoses, whereas a dictionary based on the Multilex classification system is used to code drugs. The validity of IMRD for clinical and epidemiological research has been demonstrated in previous studies.^17,18^ The use of IMRD for research has been approved by the National Health Service (NHS) Health Research Authority for medical and public health research. The scientific review committee for the IMRD database and the institutional review board at Xiangya Hospital approved this study with a waiver of informed consent. According to UK regulations, studies using fully anonymized data do not require informed consent. This study followed the Strengthening the Reporting of Observational Studies in Epidemiology (STROBE) reporting guideline for cohort studies.

### Study Design and Cohort Definition

Participants aged 18 to 89 years who underwent primary hip or knee replacement surgery between January 1, 2000, and June 30, 2023, and had at least 1 year of continuous enrollment with GPs before study entry were included. We defined joint replacement using Read codes based on previous studies using IMRD.^5,19^ Among them, we identified initiators of antiobesity medications (ie, orlistat, sibutramine, glucagon-like peptide-1 [GLP-1] receptor agonists, and rimonabant)^20,21^ based on the first prescription record of these medications after joint replacement. The date of the first antiobesity prescription was assigned as the index date for each participant. Participants were excluded if they had a history of cancer, hip or knee replacement revision, bariatric surgery before the index date, lack of body mass index (BMI; calculated as weight in kilograms divided by height in meters squared) measurement, or a BMI less than 30 at the nearest measurement before the index date.

**Figure 1.**
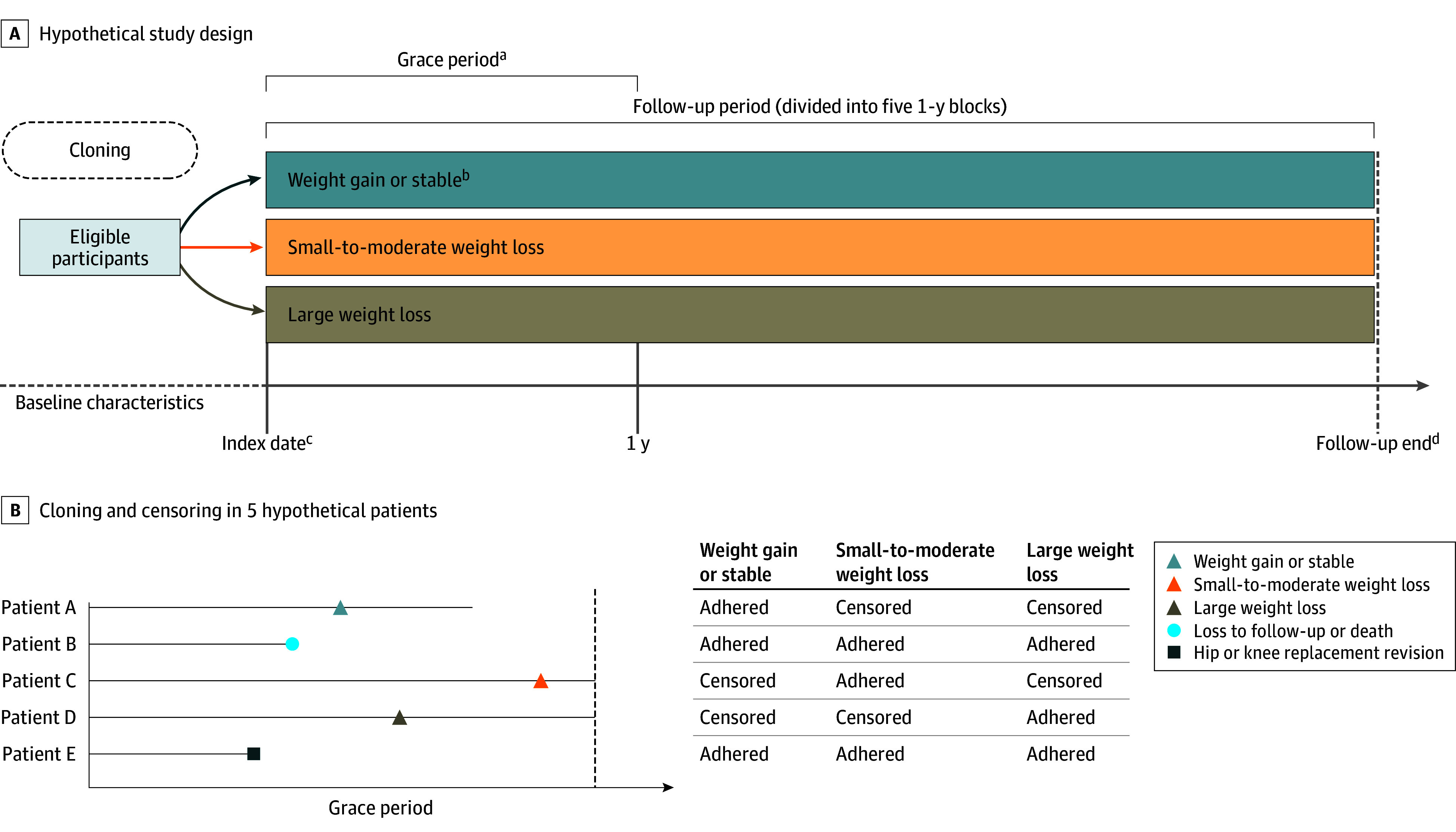
Study Design of Emulated Target Trial, and Cloning and Censoring in 5 Hypothetical Patients A, Study design of a hypothetical randomized clinical trial (target trial) on which we modeled the observational data analysis was modeled. B, Cloning and censoring in 5 hypothetical patients. ^a^Grace period: participants were given 1 year to reduce their weight after initiating with the antiobesity drugs. Clones would be censored if they deviated from their assigned treatment at the end of the grace period. ^b^Weight change = ([weight of last record during the grace period − weight at baseline]/weight at baseline × 100%). We used the following cut points to assign categories of weight change: weight gain or stable (weight loss <2% or weight gain), small-to-moderate weight loss (weight loss ≥2% or <10%), or large weight loss (weight loss ≥10%). ^c^Index date: the date of antiobesity drug initiation. ^d^Follow-up end: the date of hip or knee replacement revision, death, disenrollment from a general practitioner practice participating in IQVIA Medical Research Database, 5 years of follow-up, or the end of the study (June 30, 2023), whichever occurred first.

We emulated analyses of a hypothetical target trial using a cloning, censoring, and weighting approach^22,23,24^ to assess the association of the proportion of weight change with the risk of joint replacement revision within 1 year following the initiation of antiobesity medications (eTable 1 in Supplement 1 summarizes all protocol components from the target trial and its emulation). Specifically, the proportion of weight change was calculated by subtracting the baseline weight from the latest weight measured within 1 year after the initiation of antiobesity medication, then dividing it by the baseline weight (weight change = [weight of last record during the grace period − weight at baseline]/weight at baseline × 100%). Then, we created 3 copies for each initiator at baseline and assigned the 3 replicates to 1 of 3 weight change intervention groups (weight gain or stable [weight loss <2% or weight gain], small-to-moderate weight loss [weight loss ≥2% and <10%], or large weight loss [weight loss ≥10%]) within 1 year after initiation of antiobesity medications (Figure 1A).^25,26,27^

We allowed each copy a 1-year grace period to reach the target weight change after initiating antiobesity medication. Replicates were censored if they deviated from the assigned group within the 1-year grace period. If a participant underwent joint replacement revision before achieving the target weight change (eg, Patient E in Figure 1B), they were considered to have adhered to their assigned treatment across all replicates. In such cases, the joint replacement revision outcome was counted for each assigned group (or copies). Because censoring may lead to potential selection bias, we used inverse probability weights (IPW) to account for censoring.^23^ The denominator of the IPW was the probability that a replicate adhered to his or her assigned group using the logistic regression, which consisted of the baseline covariates and the time-varying covariates (ie, lifestyle factors, comorbidities, medication use, and health care utilization) between the index date and the date of censoring. A detailed description of the approach is provided in the eMethods in Supplement 1.

### Assessment of Outcomes

The outcomes included hip or knee replacement revision occurring within the 5 years following the index date. We defined joint replacement revision using Read codes as per previously published studies.^5,28,29^

### Assessment of Covariates

We assessed a range of covariates, including sociodemographic factors (age, sex, and socioeconomic deprivation index score), BMI, and weight, measured at baseline. We also considered lifestyle factors, such as smoking and drinking status, and comorbidities (eg, hypertension, diabetes, liver disease), which were assessed before the index date. The duration since the joint replacement was calculated as the difference between the index date and the date of the joint replacement. Medication use (eg, nonsteroidal anti-inflammatory drugs, opioids, antihypertensive drugs) during the year before the index date was also included as a covariate. Finally, health care utilization, including the number of hospitalizations, general practice visits, and referrals from specialists, was evaluated in the same year before the index date.

### Statistical Analysis

Participants were followed up until the earliest of the following events: hip or knee replacement revision, death, disenrollment from a GP practice participating in IMRD, 5 years of follow-up, or the end of the study (June 30, 2023). We divided the follow-up time into 5 1-year time blocks, starting from antiobesity medication initiation. To control for the competing risk of death, we performed a cross-sectional pooling (CSP) analysis to estimate the hazard ratios (HRs) and their corresponding 95% CI,^30^ including an indicator for the proportion of weight loss and adjusting for the year of follow-up (linear and quadratic term) and baseline covariates in the weighted population.^31^ The CSP approach models the instantaneous risk of an event using a hazard function, which preserves temporal resolution and maximizes the use of available data. We used the sandwich method to generate robust standard errors to calculate a 95% CI for HR.^32,33^ We estimated the absolute risk difference of joint replacement revision over 5 years by fitting the cross-sectional pooling models with product terms between the proportion of weight loss indicator and the year of follow-up variables. We used a nonparametric bootstrap analysis with 500 samples to compute the 95% CI for absolute estimates. Moreover, we conducted separate analyses for hip and knee replacement revision among patients with obesity and those who had undergone joint replacement surgery.

We conducted 2 secondary analyses to assess the robustness of the study findings. First, to investigate the association of the proportion of weight loss after initiating antiobesity medication with the risk of joint replacement revision, we analyzed patients with obesity and joint replacement surgery attributed to OA. Second, we adopted a similar approach for each outcome to investigate the association between the proportion of postoperative weight loss after initiating antiobesity medications and the risk of 10-year revision.

All analyses were conducted using SAS software version 9.4 (SAS Institute Inc) from October 2023 to June 2024. Two-sided *P* ≤ .05 was considered statistically significant for all tests.

## Results

**Figure 2.**
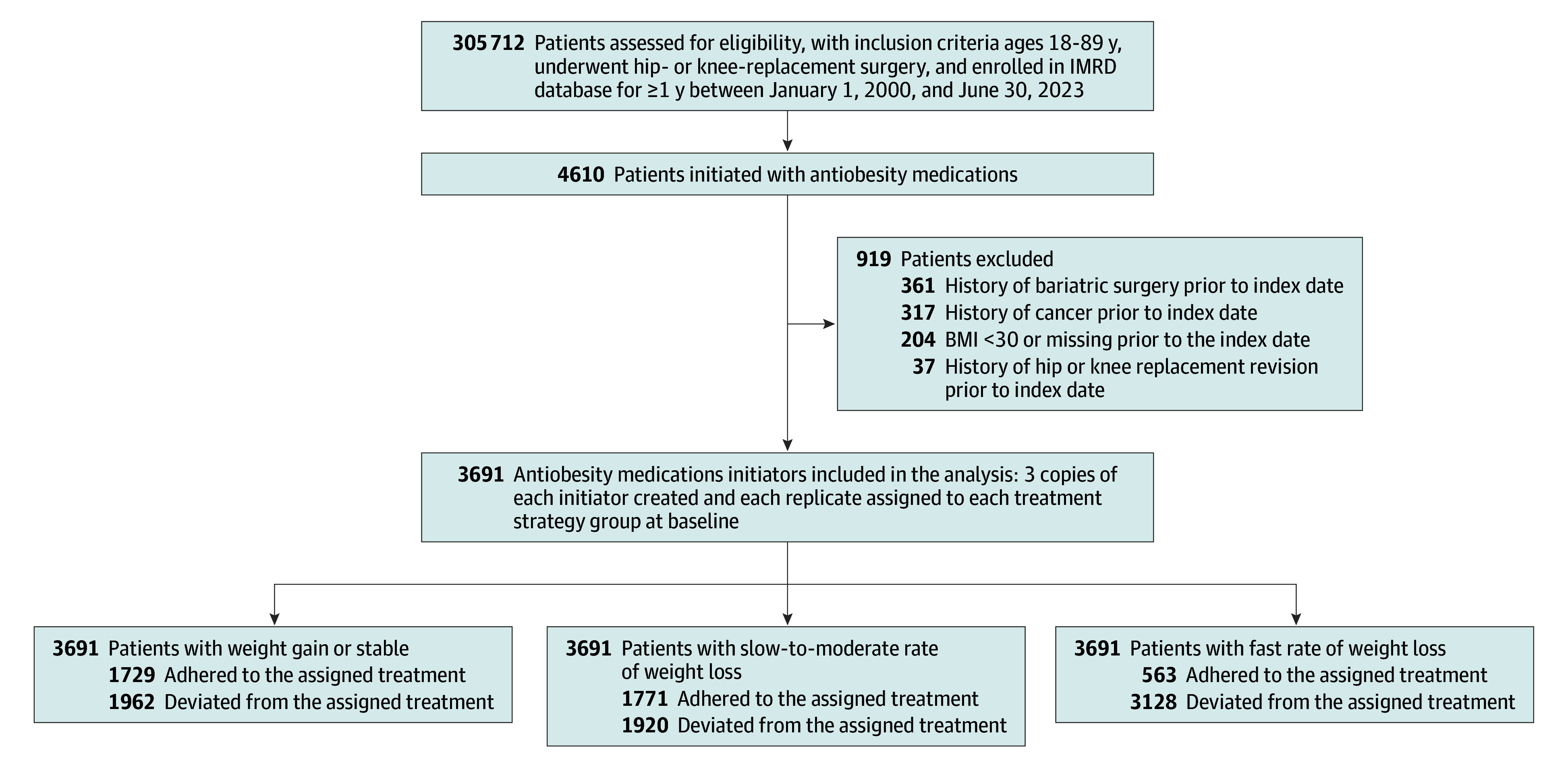
Selection Process of the Included Participants BMI indicates body mass index (calculated as weight in kilograms divided by height in meters squared); IMRD, IQVIA Medical Research Database.

The present analysis included 3691 eligible participants (Figure 2). The mean (SD) age was 64.7 (9.3) years, with 2322 (62.9%) being female. The mean (SD) BMI was 37.6 (5.1); mean (SD) weight was 104.0 (17.7) kg (Table 1).

**Table 1.** Baseline Characteristics of Patients With Obesity Initiating Antiobesity Medications

Characteristics	Study population (N = 3691)^a^
Demographics	
Age, mean (SD), y	64.7 (9.3)
Socioeconomic deprivation index, mean (SD)^b^	2.7 (1.5)
Sex	
Female	2322 (62.9)
Male	1369 (37.1)
Post joint replacement duration, mean (SD), y^c^	5.9 (5.1)
BMI, mean (SD)	37.6 (5.1)
Weight, mean (SD), kg	104.0 (17.7)
Lifestyle factors	
Drinking	
None	888 (24.1)
Past	139 (3.8)
Current	2436 (66.0)
Missing	228 (6.1)
Smoking	
None	1871 (50.7)
Past	1422 (38.5)
Current	360 (9.7)
Missing	38 (1.1)
Comorbidity	
Hypertension	2334 (63.2)
Diabetes	1480 (40.1)
Liver disease	155 (4.2)
Transient ischemic attacks	90 (2.4)
Congestive heart failure	156 (4.2)
Myocardial infarction	211 (5.7)
Pneumonia or infection	310 (8.4)
Dementia	12 (0.3)
Depression	679 (18.4)
Chronic obstructive pulmonary disease	242 (6.6)
Fall	515 (13.9)
Rheumatoid arthritis	137 (3.7)
Gastrointestinal bleeding	95 (2.6)
Stroke	111 (3.0)
Venous thromboembolism	321 (8.7)
Varicose veins	463 (12.5)
Osteoporosis	174 (4.7)
Atrial fibrillation	201 (5.4)
Osteoarthritis	2761 (74.8)
Chronic kidney disease	399 (10.8)
Anxiety	554 (15.0)
Fracture	1158 (31.4)
Medication^c^	
NSAIDs	2416 (65.5)
Opioids	1393 (37.7)
Antihypertensive medicine	2731 (73.9)
Antidiabetic medicine	1309 (35.4)
Nitrates	309 (8.4)
Estrogen	259 (7.0)
Aspirin	1091 (29.6)
Thiazide diuretics	968 (26.2)
Loop diuretics	819 (22.2)
Potassium-sparing diuretics	201 (5.4)
Proton pump inhibitors	1682 (45.6)
Glucocorticoids	1081 (29.3)
Anticoagulants	225 (6.1)
Health care utilization, mean (SD)^d^	
Hospitalizations	0.5 (1.1)
General practice visits	7.9 (6.5)
Specialist referrals	0.7 (1.2)

Abbreviations: BMI, body mass index (calculated as weight in kilograms divided by height in meters squared); NSAIDs, nonsteroidal anti-inflammatory drugs.

^a^ Unless otherwise indicated, data are expressed as No. (%) of patients.

^b^ The Socio-Economic Deprivation Index was measured by the Townsend Deprivation Index, which was grouped into quintiles from 1 (least deprived) to 5 (most deprived).

^c^ The post joint replacement duration was calculated by the year of index date minus the year of joint replacement.

^d^ Frequency during the past year.

**Figure 3.**
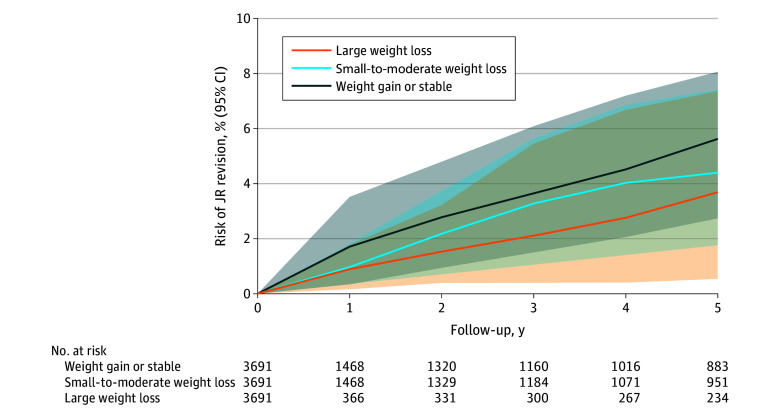
Five-Year Risk of Joint Replacement Revision Between Weight Gain or Stable, Small-to-Moderate Weight Loss, and Large Weight Loss Among Patients with Obesity and Joint Replacement (JR) Initiating Antiobesity Medications The number at risk refers to the number of replicates still being observed and at risk of experiencing the event of JR revision.

Out of the 3691 replicates assigned to each of the weight gain or stable group, small-to-moderate weight loss group, or large weight loss group, 1729, 1771, and 563, respectively, adhered to their assigned proportion of weight loss within 1 year after initiation of antiobesity medications. As shown in Figure 3, the 5-year risks of hip or knee replacement revision was 5.6% for weight gain or stable, 4.4% for small-to-moderate weight loss, and 3.7% for large weight loss group. Compared with the weight gain or stable group, the 5-year risk difference for joint replacement revision was −1.2% (95% CI, −2.6% to 0.2%) for the small-to-moderate weight loss group and −1.9% (95% CI, −3.1% to –0.7%) for the large weight loss group (Table 2). The corresponding HRs for joint replacement revision were 0.75 (95% CI, 0.55-1.04) and 0.57 (95% CI, 0.36-0.91), respectively. Consistent findings were observed when assessing the association of weight loss with the 10-year risks of joint replacement revision after initiating the antiobesity medications. Secondary analyses conducted among patients with obesity and hip or knee replacement surgery attributed to OA did not change the results materially (Table 2).

**Table 2.** Weight Loss After Initiating Antiobesity Medications and Joint Replacement Revision in Patients With Obesity

	Weight gain or stable^a^	Weight loss^a^	
		Small-to-moderate	Large
No.	3691	3691	3691
Weighted joint replacement revision, No.	168	125	86
Weighted risk over 5 y, %	5.6	4.4	3.7
Weighted risk difference over 5 y, % (95% CI)	[Reference]	−1.2 (−2.6 to 0.2)	−1.9 (−3.1 to −0.7)
Weighted HR over 5 y (95% CI)	1 [Reference]	0.75 (0.55 to 1.04)	0.57 (0.36 to 0.91)
Weighted HR over 10 y (95% CI)	1 [Reference]	0.82 (0.62 to 1.09)	0.56 (0.35 to 0.89)
Patients with OA			
No.	2761	2761	2761
Weighted joint replacement revision, No.	127	93	67
Weighted risk over 5 y, %	5.9	4.3	4.2
Weighted risk difference over 5 y, % (95% CI)	[Reference]	−1.6 (−3.1 to −0.1)	−1.7 (−3.6 to 0.2)
Weighted HR over 5 y (95% CI)	1 [Reference]	0.69 (0.48 to 0.99)	0.61 (0.35 to 1.06)
Weighted HR over 10 y (95% CI)	1 [Reference]	0.84 (0.60 to 1.17)	0.66 (0.38 to 1.16)

Abbreviations: HR, hazard ratio; OA, osteoarthritis.

^a^ Weight gain or stable: weight loss <2% or weight gain; small-to-moderate weight loss: weight loss ≥2% and <10%; large weight loss: weight loss ≥10%.

Among the 2129 participants with obesity and knee replacement assigned to each of the weight gain or stable, small-to-moderate weight loss, and large weight loss groups, 1018, 1008, and 311, respectively, adhered to their assigned treatment within 1 year after initiating antiobesity therapy. The 5-year risk of knee replacement revision was higher for the weight gain or stable group (4.5%) compared with the small-to-moderate weight loss group (2.8%; difference, −1.7 [95% CI, −2.7 to −0.7] percentage points) and the large weight loss group (2.7%; difference, −1.8 [95% CI, −3.3 to −0.3] percentage points) (eFigure 1 and eTable 2 in Supplement 1). Compared with the weight gain or stable group, the HRs for knee replacement revision were 0.55 (95% CI, 0.32-0.93) for the small-to-moderate weight loss group and 0.49 (95% CI, 0.25-0.97) for the large weight loss group. The corresponding HRs for 10-year revision were 0.67 (95% CI, 0.42-1.06) and 0.57 (95% CI, 0.27-1.21) in patients undergoing knee replacement (eTable 2 in Supplement 1).

Similar results were observed when the analysis was conducted among patients with obesity and hip replacement. Compared with the weight gain or stable group, the 5-year risk of hip replacement revision was not statistically significantly lower in the small-to-moderate weight loss group (HR, 0.82 [95% CI, 0.54-1.25]) but was statistically significantly lower in the large weight loss group (HR, 0.53 [95% CI, 0.30-0.93]) (eFigure 2 and eTable 3 in Supplement 1). The results for the 10-year risk of hip replacement revision mirrored these findings, with HRs of 0.92 (95% CI, 0.63-1.33) and 0.52 (95% CI, 0.29-0.93) for the small-to-moderate weight loss group and large weight loss group, respectively, compared with the weight gain or stable group (eTable 3 in Supplement 1).

## Discussion

In this cohort study of a large UK GP electronic health records database, a higher proportion of weight loss within 1 year after initiating antiobesity medications was associated with a lower risk of 5- and 10-year revision among patients with obesity undergoing joint replacement. Despite clinical guidelines advocating weight management before joint replacements,^34^ the proportion of individuals who are classified as obese has reached more than 50% in patients undergoing hip replacement and 70% in patients undergoing knee replacement.^35^ However, little information is available to guide postoperative optimization of weight loss management for this population. Only one study investigated postoperative weight loss and risk for revision after joint replacement, and they found that at least 5% BMI loss postoperatively was not associated with the risk for all-cause revisions.^36^ Nevertheless, the reason for the weight loss was unclear in this study, and the mean follow-up was relatively short. We observed that small-to-moderate (2%-10%) and large (≥10%) weight loss over 1 year after initiating antiobesity medications were associated with a 25% and 43% lower risk of 5-year revision, respectively, compared with weight gain or stable (<2%). This suggests that weight loss after initiating antiobesity medication improves implant survivorship for joint replacements.

### Possible Explanations

Several explanations have been proposed for the association between postoperative weight loss and a decreased risk of revision following joint replacements. First, previous studies have demonstrated that revision in patients with obesity are significantly more likely than in control patients due to aseptic loosening and mechanical complications in joint replacements,^37,38,39,40^ and the increased stress placed on joint components and fixation interface in patients with excess body mass could lead to the increased likelihood of mechanical joint failure.^41^ In the current study, the observed reduction in revision rates may be partially attributed to decreased mechanical stress on the joint following weight loss induced by antiobesity medications. Second, in addition to reduced BMI, weight loss after initiating antiobesity medication could also provide additional benefits to obesity-related comorbidities, including type 2 diabetes and hypertension, which is associated with increased revision in patients undergoing joint replacement.^42,43,44^ Accordingly, the mitigation of these comorbidities through postoperative weight loss could further explain the reduced risk of revision, particularly revisions related to infections.

### Clinical and Research Implications

The 2023 ACR/AAHKS Clinical Practice Guideline developed a consensus that obesity alone should not be a barrier to joint replacement.^16^ The high obesity rate in patients undergoing joint replacement is expected to increase. Given that postoperative weight loss could have potential benefits, it is important to adopt strategies to lose weight for patients with obesity who underwent joint replacement. Unfortunately, less than 15% of patients experience substantial weight loss after joint replacement.^45,46,47^ Although weight loss by lifestyle intervention has been shown to provide substantial benefits for the obese population, modification of diet and exercise alone is unlikely to result in long-term benefits due to difficulty maintaining the intervention.^48^ Pharmacotherapy, with relatively safe, effective, and sustainable weight loss, could be considered another strategy for obesity management.^9^ Our results provide evidence that 2% or more weight loss over 1 year after initiating antiobesity medications reduces the 5- and 10-year risk of revision for joint replacements. If future studies confirm these findings, they could guide clinical practice and improve implant survivorship of joint replacements in the obese population.

### Strengths and Limitations

Several strengths of our study are noteworthy. First, although we could theoretically design a randomized clinical trial (RCT) to examine the effect of different proportions of weight loss within 1 year after initiating antiobesity medications on the risk of revision following joint replacements, several methodological and logistic difficulties make an RCT challenging. The cost of conducting such a trial, which would entail recruiting over 3500 individuals with obesity who have undergone hip or knee replacement and following them for 5-10 years, would be substantial. Additionally, logistically, ensuring each participant achieves the designated target proportion of weight loss within the first year after initiating antiobesity medication would be arduous. Moreover, there would be an ethical concern about including a treatment arm in the RCT where participants are required to experience weight gain or stable over the course of a year after initiating antiobesity medication. Using data from the observational study, we emulated a target clinical trial to assess the association of weight loss proportion after initiating antiobesity medication with the risk of joint revision. We followed several principles of clinical trials, including a clearly-defined intervention strategy (ie, 3 groups of weight loss: <2%, 2%-10%, and >10%) over 1 year using the antiobesity medications (ie, orlistat, sibutramine, GLP-1 receptor agonists, and rimonabant), a clearly-defined target population (ie, patients with obesity undergoing joint replacement), and well-defined study outcomes (ie, revision following joint replacements). Second, we started the follow-up from the date of initiation of antiobesity medications to avoid potentially immortal time bias. Additionally, we adjusted for potential confounders and used inverse probability weighting to account for the loss to follow-up. All these measures enhanced the internal validity of the study findings.

This study also has limitations. First, although we used rigorous approaches to control confounders, some covariates, such as surgical and implant-related factors, may need to be better captured by the variables available in IMRD; thus, we cannot rule out residual confounding. Second, the IMRD does not contain hospitalization data, and the reasons for revision were not obtained. We cannot further analyze which kind of revision was reduced by postoperative weight loss. Third, our study estimated weight change using baseline BMI for 19.3% of participants who did not have follow-up measurements. However, this approach allowed us to retain a larger sample size, ensuring broader generalizability of our findings. Fourth, we included all patients undergoing hip or knee replacement rather than restricting the analysis to those with OA alone, which may introduce variability in the outcomes. Fifth, we cannot assess the association of weight loss from a specific antiobesity medication with the risk of revision of joint replacement owing to study power. However, to our knowledge, there is no evidence suggesting that a particular antiobesity medication has a direct effect on the risk of revision. Additionally, because of the limitation of follow-up data in the IMRD database, it cannot evaluate the effect of postoperative weight loss on the longer-term revision rate, such as 15 or 20 years.

## Conclusions

In this cohort study emulating an RCT, a higher proportion of weight loss after initiating antiobesity medications within 1 year was associated with a lower risk of 5-year and 10-year revision among patients with obesity undergoing joint replacement. These results suggest that antiobesity medication use, with relatively safe, effective, and sustainable weight loss, may be a good strategy for improving implant survivorship among patients with obesity undergoing hip or knee replacement.

## Notes

Supplement 1.
eMethods.
**eTable 1.** Protocol of a Target Trial and an Emulated Trial Using Observational Data **eTable 2.** Relations of Weight Loss After Initiating Anti-Obesity Medications to Knee Replacement Revision in Obese Patients **eTable 3.** Relations of Weight Loss After Initiating Anti-Obesity Medications to Hip Replacement Revision in Obese Patients **eFigure 1.** 5-Year Risk of Knee Replacement Revision Between Weight Gain or Stable, Small-to-Moderate Weight Loss, and Large Weight Loss Among Patients With Obesity and Knee Replacement Initiating Anti-Obesity Medications **eFigure 2.** 5-Year Risk of Hip Replacement Revision Between Weight Gain or Stable, Small-to-Moderate Weight Loss, and Large Weight Loss Among Patients With Obesity and Hip Replacement Initiating Anti-Obesity Medications

Supplement 2.
Data Sharing Statement
