# Supplementary material for: Postoperative Weight Loss After Antiobesity Medications and Revision Risk After Joint Replacement
Source: JAMA Netw Open. 2025 Feb 21;8(2):e2461200. doi: 10.1001/jamanetworkopen.2024.61200 (PMC11846009; doi:10.1001/jamanetworkopen.2024.61200)
Supplement: Supplement 1. — eMethods. eTable 1. Protocol of a Target Trial and an Emulated Trial Using Observational Data eTable 2. Relations of Weight Loss After Initiating Anti-Obesity Medications to Knee Replacement Revision in Obese Patients eTable 3. Relations of Weight Loss After Initiating Anti-Obesity Medications to Hip Replacement Revision in Obese Patients eFigure 1. 5-Year Risk of Knee Replacement Revision Between Weight Gain or Stable, Small-to-Moderate Weight Loss, and Large Weight Loss Among Patients With Obesity and Knee Replacement Initiating Anti-Obesity Medications eFigure 2. 5-Year Risk of Hip Replacement Revision Between Weight Gain or Stable, Small-to-Moderate Weight Loss, and Large Weight Loss Among Patients With Obesity and Hip Replacement Initiating Anti-Obesity Medications [file jamanetwopen-e2461200-s001.pdf]

Supplemental Online Content

Xie D, Englund M, Lane NE, et al. Postoperative weight loss after anti-obesity medications and revision risk after joint replacement. *JAMA Netw Open.* 2025;8(2):e2461200. doi:10.1001/jamanetworkopen.2024.61200

eMethods

eTable 1. Protocol of a Target Trial and an Emulated Trial Using Observational Data

eTable 2. Relations of Weight Loss After Initiating Anti-Obesity Medications to Knee Replacement Revision in Obese Patients

eTable 3. Relations of Weight Loss After Initiating Anti-Obesity Medications to Hip Replacement Revision in Obese Patients

eFigure 1. 5-Year Risk of Knee Replacement Revision Between Weight Gain or Stable, Small-to-Moderate Weight Loss, and Large Weight Loss Among Patients With Obesity and Knee Replacement Initiating Anti-Obesity Medications

eFigure 2. 5-Year Risk of Hip Replacement Revision Between Weight Gain or Stable, Small-to-Moderate Weight Loss, and Large Weight Loss Among Patients With Obesity and Hip Replacement Initiating Anti-Obesity Medications

This supplemental material has been provided by the authors to give readers additional information about their work.

© 2025 Xie D et al. *JAMA Network Open.*

eMethods

Detail information of the study design: “cloning, censoring, and weighting”

Cloning: assigning patients to treatment groups at time zero

We generated three replicates for each participant and assigned them to one of three intervention groups based on their weight change within the first year after initiating anti-obesity medications: (1) weight gain or stable (weight loss <2% or weight gain), (2) small-to-moderate weight loss (2%≤ weight loss <10%), or (3) large weight loss (weight loss ≥10%). This strategy closely resembles the randomization process used in traditional RCTs.

Censoring: ensuring adherence to assigned treatment strategy during the first-year follow-up

We allowed each replicate a 1-year grace period to reach the target weight change after initiating anti-obesity medication. Replicates were censored if they deviated from their assigned treatment during this period. For example, if a participant lost less than 2% of their weight or gained weight within the first year after initiating anti-obesity medications (e.g., Patient A in **Figure 1B**), the copy of the participant assigned to the weight gain or stable intervention arm would remain adherent and would not be censored. However, the two other copies of Patient A, assigned to the small-to-moderate or large weight loss intervention arms, deviated from their assigned groups and would be censored based on their weight change. Therefore, the joint replacement revision outcome was only counted for the replicate in the weight gain or stable treatment group. If a participant underwent joint replacement revision before achieving the target weight change (e.g., Patient E in **Figure 1B**), they were considered to have adhered to their assigned treatment across all replicates. In such cases, the joint replacement revision outcome was counted for each assigned group (or clones).

Weighting: addressing selection bias from censoring

To address potential selection bias arising from censoring, we applied inverse probability weighting (IPW). The IPW denominator was the probability of a replicate adhering to their assigned treatment, which was estimated using logistic regression that included both baseline covariates (see “**Assessment of covariates**”) and the time-varying covariates (i.e., lifestyle factors, comorbidities, medication use, and healthcare utilization).

© 2025 Xie D et al. *JAMA Network Open.*

61 **eTable 1. Protocol of a target trial and an emulated trial using observational data**

Protocol Component	Target Trial Specification	Target Trial Emulation
Source of data	Across multiple clinical trial centers or hospitals	IMRD database
Eligibility Criteria	• Aged 18-89 years<br>• Participants undergoing primary hip or knee replacement surgery	Same as for the target trial, except:<br>• Required enrollment in IMRD database for at least one year
Treatment Strategies	Treatment with one of the following categories:<br>(1) Weight gain or stable: weight loss < 2% or weight gain within one year after initiation of anti-obesity medications<br>(2) Small-to-moderate weight loss: 2% ≤ weight loss < 10% within one year after initiation of anti-obesity medications<br>(3) Large weight loss: weight loss ≥ 10% within one year after initiation of anti-obesity medications	Same as for the target trial.
Treatment Assignment	Participants are randomly assigned to a treatment group at initiation.	Same as for the target trial, except we created three copies of each individual (clones) and assigned each of the clones to one of the treatment strategies at initiation.
Outcomes	Hip or knee replacement revision	Same as for the target trial.
Follow-up	Follow-up for each individual starts on the date of anti-obesity prescription initiation and ends on the date of hip or knee replacement revision, death, loss to follow-up, or administrative end of follow-up, whichever occurs first.	Same as for the target trial.
Causal Contrasts	Intention-to-treat effect and per-protocol effect.	Observational analog of the per-protocol effect.
Statistical Analysis	Intention-to-treat analysis: Kaplan-Meier estimator to construct survival curves and a Cox regression model to estimate a hazards ratio.<br>Per-protocol analysis: same as the intention-to-treat analysis except that individuals are artificially censored when they deviate from the assigned treatment strategy. Inverse probability weighting is used to	Same as the target trial except that duplication of individuals is used to address unknown baseline treatment assignments in the observational dataset.

© 2025 Xie D et al. *JAMA Network Open*.

	adjust for potential selection bias due to artificial censoring.	

62 Abbreviations: IMRD, IQVIA Medical Research Database; GP, General Practitioner.

© 2025 Xie D et al. *JAMA Network Open*.

eTable 2. Relations of weight loss after initiating anti-obesity medications to knee replacement revision in obese patients

	Weight gain or stable[a]	Small-to-moderate weight loss[a]	Large weight loss[a]
Number	2,129	2,129	2,129
Weighted knee replacement revision (n)	79	49	27
Weighted risk over 5 years (%)	4.5	2.8	2.7
Weighted risk difference over 5 years (%, 95% CI)	0.0 (reference)	-1.7 (-2.7 to -0.7)	-1.8 (-3.3 to -0.3)
Weighted HR over 5 years (95% CI)	1.00 (reference)	0.55 (0.32 to 0.93)	0.49 (0.25 to 0.97)
Weighted HR over 10 years (95% CI)	1.00 (reference)	0.67 (0.42 to 1.06)	0.57 (0.27 to 1.21)

Abbreviations: HR, hazard ratio; n, number; 95% CI, 95% confidence interval.

[a] Weight gain or stable: weight loss <2% or weight gain; Small-to-moderate weight loss: 2%≤ weight loss <10%; Large weight loss: weight loss ≥10%.

© 2025 Xie D et al. *JAMA Network Open*.

eTable 3. Relations of weight loss after initiating anti-obesity medications to hip replacement revision in obese patients

	**Weight gain or stable[a]**	**Small-to-moderate weight loss[a]**	**Large weight loss[a]**
Number	1,728	1,728	1,728
Weighted hip replacement revision (n)	84	73	49
Weighted risk over 5 years (%)	6.0	5.4	4.1
Weighted risk difference over 5 years (%, 95% CI)	0.0 (reference)	-0.6 (-2.6 to 1.4)	-1.9 (-3.5 to -0.3)
Weighted HR over 5 years (95% CI)	1.00 (reference)	0.82 (0.54 to 1.25)	0.53 (0.30 to 0.93)
Weighted HR over 10 years (95% CI)	1.00 (reference)	0.92 (0.63 to 1.33)	0.52 (0.29 to 0.93)

Abbreviations: HR, hazard ratio; n, number; 95% CI, 95% confidence interval.

[a] Weight gain or stable: weight loss <2% or weight gain; Small-to-moderate weight loss: 2%≤ weight loss <10%; Large weight loss: weight loss ≥10%.

© 2025 Xie D et al. *JAMA Network Open*.

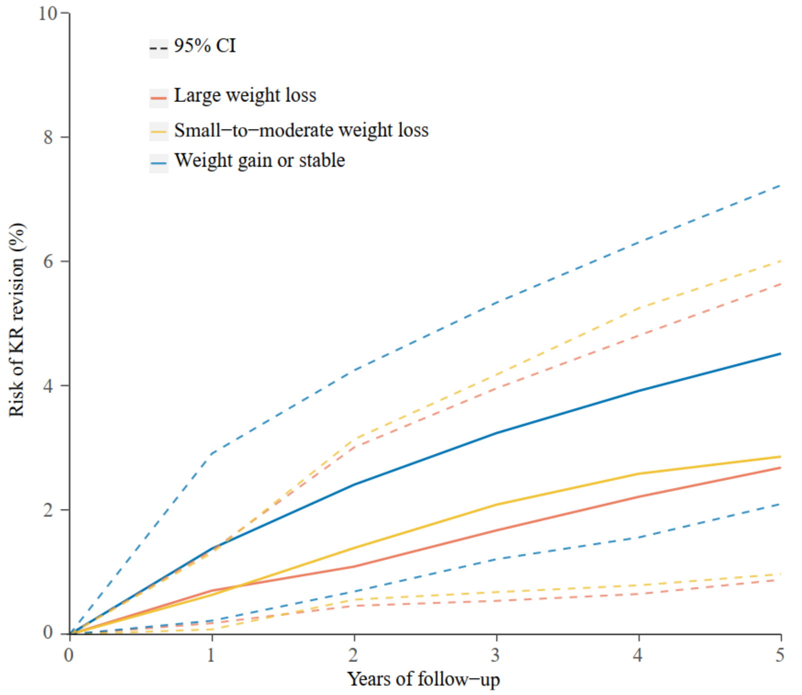

Number at risk						
**Weight gain or stable**	2129	863	767	666	585	501
**Small-to-moderate weight loss**	2129	838	759	676	609	534
**Large weight loss**	2129	200	177	160	140	118

eFigure 1. 5-year risk of knee replacement revision between weight gain or stable, small-to-moderate weight loss, and large weight loss among patients with obesity and knee replacement initiating anti-obesity medications. The number at risk refers to the number of replicates still being observed and at risk of experiencing the event of knee replacement revision. KR, knee replacement.

© 2025 Xie D et al. *JAMA Network Open*.

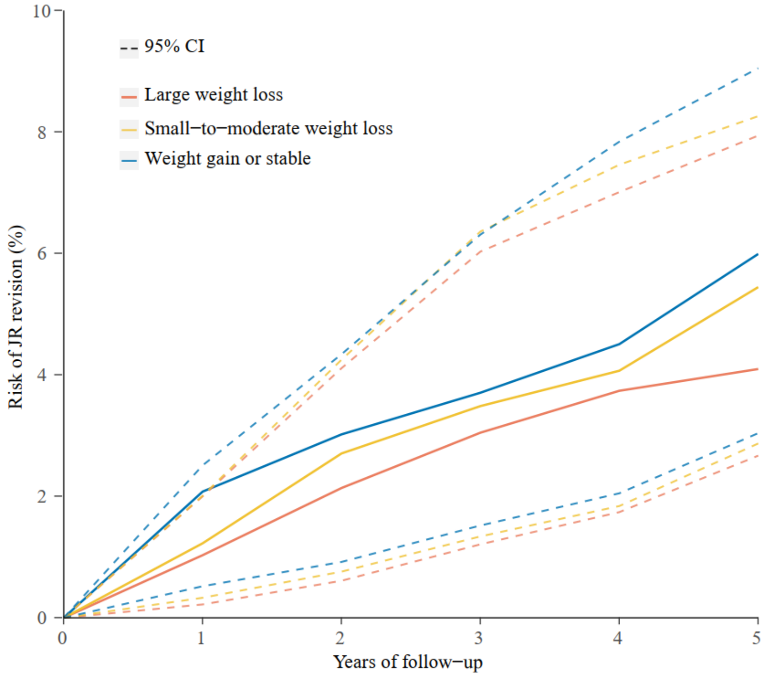

**Number at risk**						
**Weight gain or stable**	1728	669	609	545	482	425
**Small-to-moderate weight loss**	1728	693	627	562	512	458
**Large weight loss**	1728	185	173	159	144	130

eFigure 2. 5-year risk of hip replacement revision between weight gain or stable, small-to-moderate weight loss, and large weight loss among patients with obesity and hip replacement initiating anti-obesity medications. The number at risk refers to the number of replicates still being observed and at risk of experiencing the event of hip replacement revision. HR, hip replacement.

© 2025 Xie D et al. *JAMA Network Open*.
